# Laboratory risk assessment of *Beauveria bassiana* AAD16 on two species of ladybird beetle

**DOI:** 10.1371/journal.pone.0317483

**Published:** 2025-01-30

**Authors:** Md. Rajib Hasan, Md. Rasel Raju, Un Taek Lim

**Affiliations:** Department of Plant Medicals, Andong National University, Andong, Republic of Korea; Chiang Mai University Faculty of Agriculture, THAILAND

## Abstract

*Beauveria bassiana* AAD16, isolated from *Allomyrina dichotoma*, shows promise as a mycoinsecticide against various coleopterans. However, assessing non-target impacts on beneficial beetles like ladybirds is crucial before commercialization. Here we assessed the compatibility between ladybird beetles and *B*. *bassiana* AAD16. The virulence of the AAD16 strain was compared with that of an available commercial strain, *B*. *bassiana* GHA, for two developmental stages (adults and 3^rd^ instar larvae) of two coccinellids, *Harmonia axyridis* Pallas and *Chilocorus* spp. Say using the topical (1μl) application method. The ST_50_ for the two life stages of the two ladybird beetles were not different between the two tested fungal strains. Mycosis rates recorded from the dead bodies were also not significant except in the 3^rd^ instar which showed 36 and 63% from AAD16 and GHA strains in *H*. *axyridis*, while those of *Chilocorus* spp. were 40 and 63%, respectively. In adult stage, the mycosis rates of *H*. *axyridis* (males and females tested separately) were (20–23) % and (26–30) % from the AAD16 and GHA strains, while those of *Chilocorus* spp. (unsexed) were 23 and 30%, respectively. AAD16 caused similar rates of mortality in the adult stages of both species. Therefore, we conclude that *B*. *bassiana* AAD16 would not increase risk to these beneficial insects compared to a similar pathogen commercialized.

## Introduction

*Beauveria bassiana* (Balsamo) Vuillemin formulations serve as sustainable and environmentally safe insecticides, providing an alternative to chemical pesticides for controlling numerous pests in agricultural and forestry systems [1–4]. Many countries have developed and registered commercial products based on *B*. *bassiana*, which are now utilized globally for pest control [3, 5–7]. However, new molecular studies show that *B*. *bassiana*, once thought to be a general-purpose fungus, is a group of closely related species, each adapted to specific hosts or environments [2, 3]. *B*. *bassiana* is a widespread soil-dwelling entomopathogenic fungus, making it particularly effective against organisms with soil-associated life stages, such as overwintering coccinellids, melolonthine scarabs, and caterpillars [8–10]. Additionally, some *B*. *bassiana* strains have been isolated from the phylloplane (leaf surfaces) of hedgerow vegetation [11], demonstrating its ecological flexibility.

Strains of *B*. *bassiana* have been shown to cause high levels of mortality and infection in some non-target organisms [1], as well as to the targeted pests [12–18]. In contrast, other strains, such as GHA, are less harmful to non-target organisms than to their target pests [1, 19, 20]. The virulence and mycosis rates of *B*. *bassiana* differ according to the host species and the enzymatic characteristics of the strains [21].

*Harmonia axyridis* (Pallas) (Coleoptera: Coccinellidae) is a ladybird beetle native to East Asia that can become invasive on other continents. Due to its environmental resiliency and aggressive predation, it has been a very useful biological control agent for aphids, coccids, and other pests, and it has been widely used in horticulture [22]. However, natural enemies of *H*. *axyridis* have also been identified including pathogens, parasites, and parasitoids [23–25]. Despite the presence of these natural enemies of *H*. *axyridis*, the success of *H*. *axyridis* as a biological control agent of insect pests may imply the existence of biological resistance to them. *H*. *axyridis* has a robust immune system, giving it defenses that surpass competing ladybirds [26–30]. The immune system of *H*. *axyridis* consists of chemical defenses against a variety of bacteria as well as a wide range of antimicrobial peptides that are the result of multiple gene duplication events [26]. Additionally, *H*. *axyridis* possesses strong alkaloid chemical defenses against predators and pathogens, which give it a foul smell and taste.

*Chilocorus* spp. (Say) (Coleoptera: Coccinellidae) is an omnivorous predator of several scale insects, aphids, and mealybugs [31]. It has been reported often as a predator of the pine needle scale (*Chionaspis pinifoliae*) [32].

The interactions between *B*. *bassiana* and coccinellids have been explored from two primary perspectives. The first of these is the study of the mortality of overwintering populations of coccinellids that are naturally exposed *B*. *bassiana* in the soil [9, 33–35], while the second study focus has been deaths of non-target coccinellids when exposed to *B*. *bassian*a used as biorational pesticides [36–41]. No previous studies have addressed the susceptibility of *Chilocorus* spp. to any entomopathogenic fungi, and the risk to *H*. *axyridis* has only been assessed as having low susceptibility to commercial *B*. *bassiana* GHA [27]. Therefore, the objective of this study was to assess the compatibility of adults and 3^rd^ instar larvae of *H*. *axyridis* and *Chilocorus* spp. with *B*. *bassiana* AAD16 in comparison to a commercial product based on *B*. *bassiana* GHA.

## Materials and methods

### Insect colonies

Colonies of *H*. *axyridis* and *Chilocorus* spp. originated from adult beetles collected from peach orchards at the experimental fields of Andong National University, Korea (36° 34’ 6.0744’ N latitude and 128° 43’ 45.6852’’ E longitudes). The colonies were housed in onion bags (45×30 cm) in a peach tree naturally infested with aphids (*Myzus persicae* Sulzer). Onion bags, made from synthetic fabrics, permit ventilation and are used for containing field population of the ladybird species for short period of time. The 3^rd^ instar larvae and adults of both ladybird species were collected as needed from the onion bag for laboratory bioassays.

### Fungal pathogens used and preparation of the conidial suspensions tested

Two strains (AAD16 and GHA) of *B*. *bassiana* were evaluated in this study. The AAD16 strain was isolated from the scarabaeid beetle *Allomyrina dichotoma* (L.) [42], while GHA (Botanigard® ES) is a commercially available strain from Laverlam International Cooperation (Butte, Montana, USA) that was grown in our laboratory on Sabouraud Dextrose Agar (SDA) media (Difco™, Sparks, MD, USA) in Petri dishes (60 mm diameter × 15 mm height) maintained in the dark at 25.3 ± 0.1°C and 94.9 ± 0.3% RH for 14 days [43]. Strain AAD16 was also cultured in SDA media in the dark at 25°C. Conidia were collected from the culture surfaces by scraping, and then the conidia were suspended in sterile distilled water with 0.1% Triton X-100 in a 20-ml scintillation vial (240804, Wheaton, Millville, NJ) containing autoclaved Triton X-100 (0.1%) solution (Duksan Pure Chemicals Co. Ltd., Ansan, Republic of Korea). Conidial suspensions were vortexed for at least 2 minutes until it became a homogeneous suspension. The conidial concentration of the suspension was measured using a Neubauer hemocytometer (Marienfeld-Superior, Paul Marienfeld GmbH and Co. KG, Lauda-Königshofen, Germany). Conidial viability was assessed before the bioassay by spreading 100μl of 1×10^4^ CFU/ml suspension on SDA plates. The plates were incubated at 25°C, and the percentage germination was determined after 18 hours for groups of 100 spores by placing a sterile microscope cover slip on each plate under a compound microscope. In all experiments, conidia germination exceeded 95%.

### Adult and larval bioassays

Two developmental stages (adults and 3^rd^ instar larvae) of two coccinellids, *H*. *axyridis* and *Chilocorus* spp., were tested using 1×10^8^ conidia/ml concentrations of both *B*. *bassiana* AAD16 and GHA strain, using 0.1% Triton X-100 ddH_2_O as a control. For *H*. *axyridis*, the two sexes of adults were tested separately; but for *Chilocorus* spp., unsexed adults were tested. On the day of the experiment, beetles were collected from the colony and placed in Petri dishes (9 cm dia) sealed with parafilm to prevent escape. The dishes were then placed on a container filled with ice for 10 minutes to reduce beetle activity before the topical application. All bioassays were done with adults and 3rd instar larvae. Beetles were removed from the ice, and 1μl of a treatment was applied using micro syringe onto the dorsal surface of the abdomen. The adult beetles were subsequently placed in sterile 9 cm-dia Petri dishes with 10 beetles/dish. All plates with the same treatment were grouped together and held in desiccators to maintain high relative humidity (4202–0000, Bel-Art Products, Pequannock, New Jersey, USA), while maintained at 25.3 ± 0.1°C and 95.1 ± 3.4% RH in an incubator. Treated larvae were kept in 2 ml Eppendorf tubes without a food source, with a small hole in the lid. Tubes were kept in desiccators (4202–0000, Bel-Art Products, Pequannock, New Jersey, USA) at 25.2 ± 0.1°C and 94.7 ± 0.4% RH inside a growth chamber.

Mortality and mycosis rates (%) were recorded for adult beetles at 24-hour intervals from the time of exposure until 23 days, and for larvae until 15 days. An insect was classified as dead when there was no movement observed following any of three strokes with a fine brush under a stereo-microscope. Insects were categorized as having mycosis by *B*. *bassiana* when fungus mycelia were visible on the insects’ integument through a stereo-microscope.

### Data analysis

All statistical analyses were conducted using SAS version 9.3 [44]. The median survival time (ST_50_) per treatment was determined, and treatment values were compared using Kaplan-Meier survival analysis since mortality did not reach 100% in either the treatment or control groups. Subsequently, the survival curves were compared using the Log-rank test at the 95% confidence level. Significant differences among treatments were determined by assessing the 95% confidence interval (CI). The fungal mycosis development rates over time within a strain of the AAD16 and GHA strains were analyzed with repeated measures ANOVA, along with post hoc multiple comparison tests that were analogous to Tukey’s test [45]. Data for mortality and fungal mycosis rates of larval stage between the AAD16 and GHA strains were compared using repeated measures ANOVA.

## Result

### Adult bioassay

**Fig 1:**
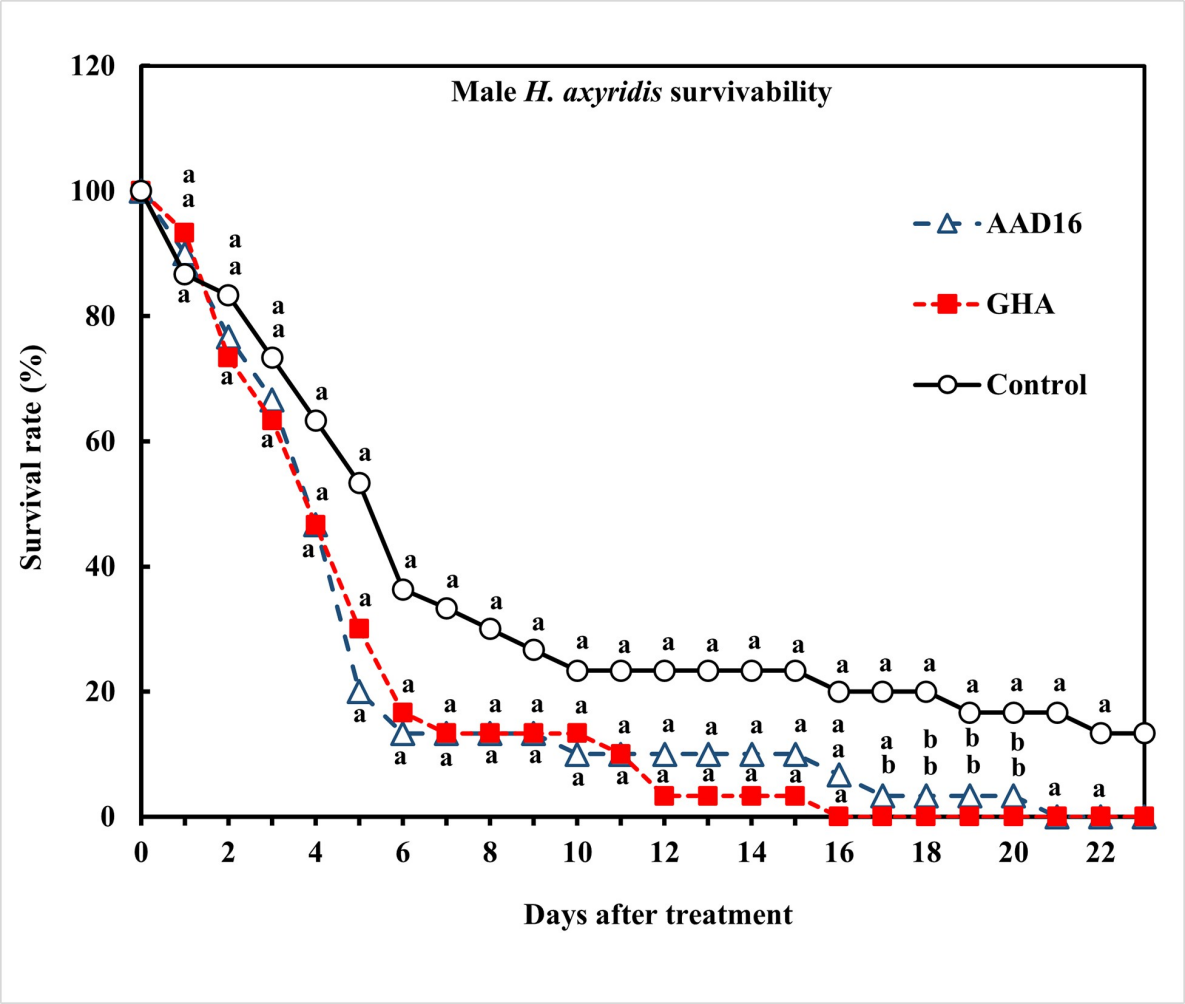
Survival (%) of adult male *H*. *axyridis* exposed to 1×10^8^ conidia/ml after exposure for 24 h to different entomopathogenic fungi. Means followed by the same letter were not significantly different from each other (Tukey studentized range HSD test, α = 0.05).

**Fig 2:**
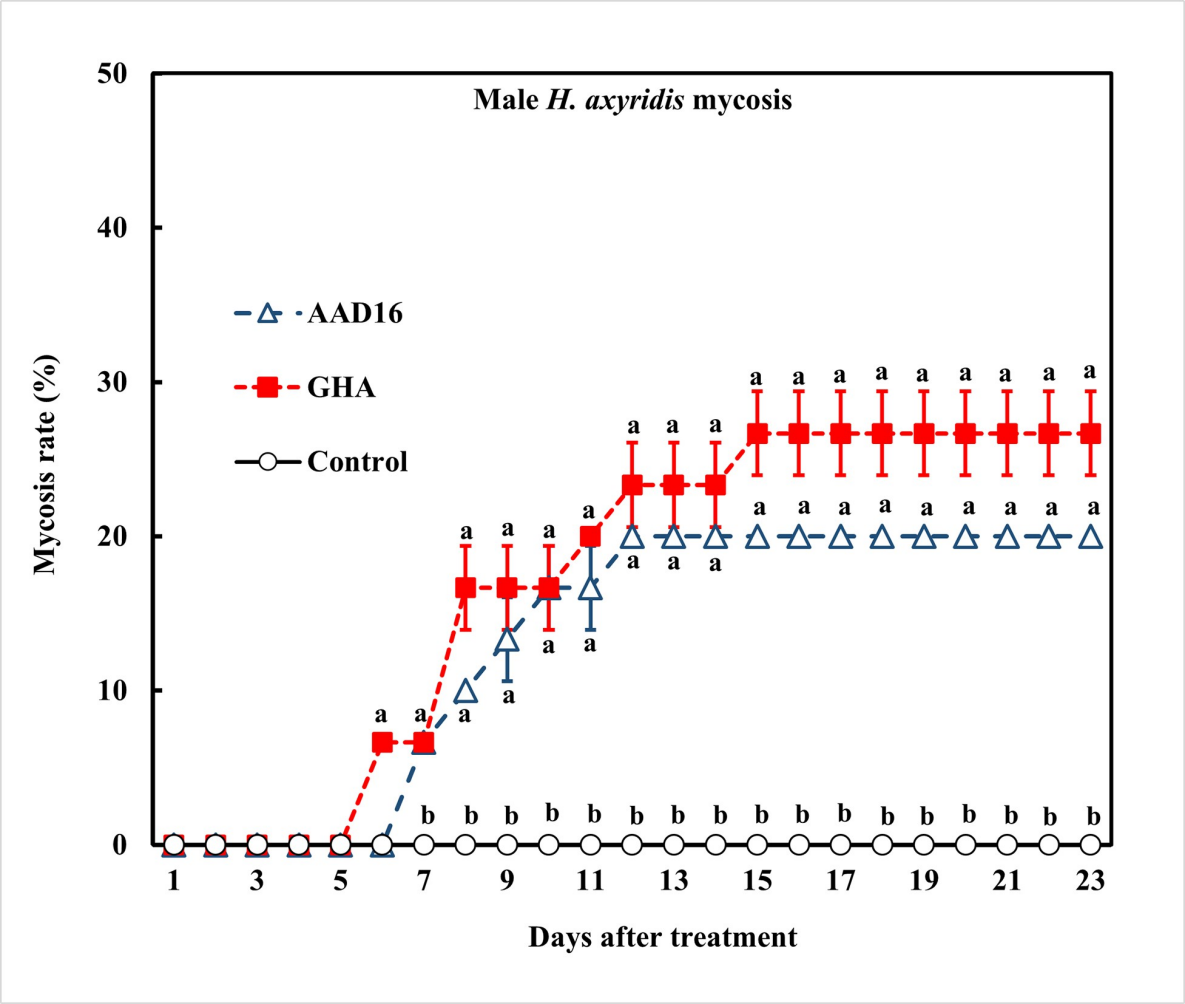
Mycosis rates for adult males of *H*. *axyridis* exposed to 1×10^8^ conidia/ml after exposure for 24 h to different entomopathogenic fungi. Means followed by the same letter are not significantly different (Tukey studentized range HSD test, α = 0.05).

For adult’s male of *H*. *axyridis*, the ST_50_ value of *B*. *bassiana* AAD16 was 80.01 h (*χ*^*2*^ = 122.97, *df* = 19, *P* < 0.001), which did not differ significantly from that of *B*. *bassiana* GHA, which had an ST_50_ value of 82.30 h (*χ*^*2*^ = 104.12, *df* = 16, *P* < 0.001) (Table 1). Survival analysis was carried out with adult males of *H*. *axyridis* exposed to entomopathogenic fungi, and survival rates were significantly different among the treatments (*χ*^*2*^ *=* 6.10, *df =* 2, *P =* 0.047) (Fig 1). The mortality rate of males was 100% for AAD16 and GHA, compared to 87% in the control over the entire period of observation (Fig 1). No significant difference in mycosis rates was observed between two fungus treatments (Treatment: *F =* 34.72, *df =* 1, 22, *P <* 0.001; Time: *F =* 49.10, *df =* 22, 92, *P <* 0.001; Interaction: *F =* 1.04, *df =* 22, 92, *P <* 0.431) (Fig 2).

**Table 1:** Comparison of fungal efficacy against adult males of *H*. *axyridis* exposed to 1×10^8^ conidia/ml at 1μl per insect via topical application method (n = 30).

Treatment	ST_50_	95% CI	Slope ± SE	*χ*^*2*^ (df)
*B*. *bassiana* AAD16	80.01a	67.66–91.80	5.26 ± 0.47	122.97 (19)
*B*. *bassiana* GHA	82.30a	70.37–93.60	5.73 ± 0.56	104.12 (16)
Control	121.78b	100.95–141.86	3.80 ± 0.41	83.00 (17)

ST_50_ values followed by the same letters are not significantly different among treatments at the 95% CI.

ST_50_, Median survival time; CI, Confidence interval.

**Fig 3:**
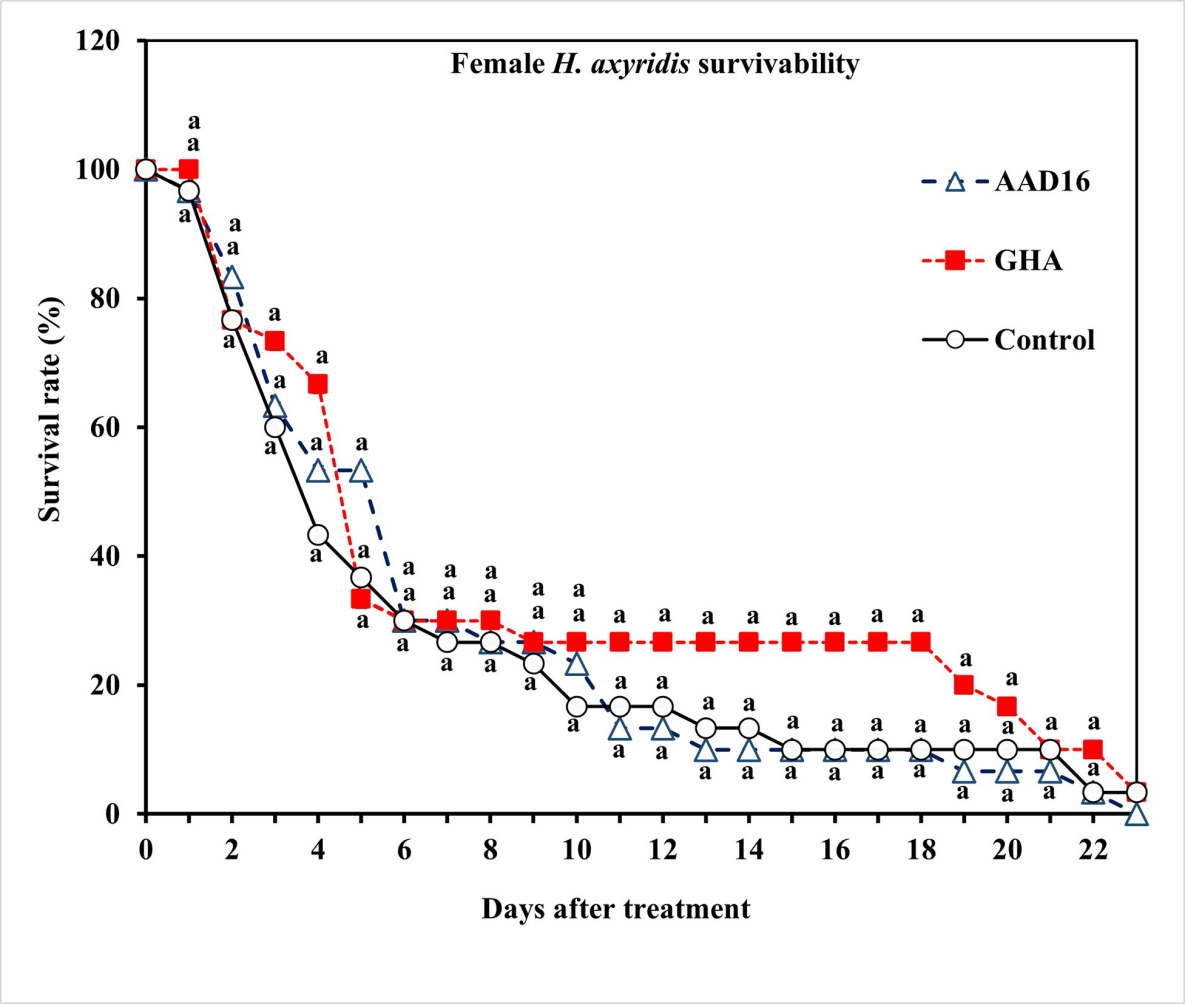
Survival (%) of adult females of *H*. *axyridis* exposed to 1×10^8^ conidia/ml after exposure for 24 h to different entomopathogenic fungi. Means followed by the same letter are not significantly different (Tukey studentized range HSD test, α = 0.05).

**Fig 4:**
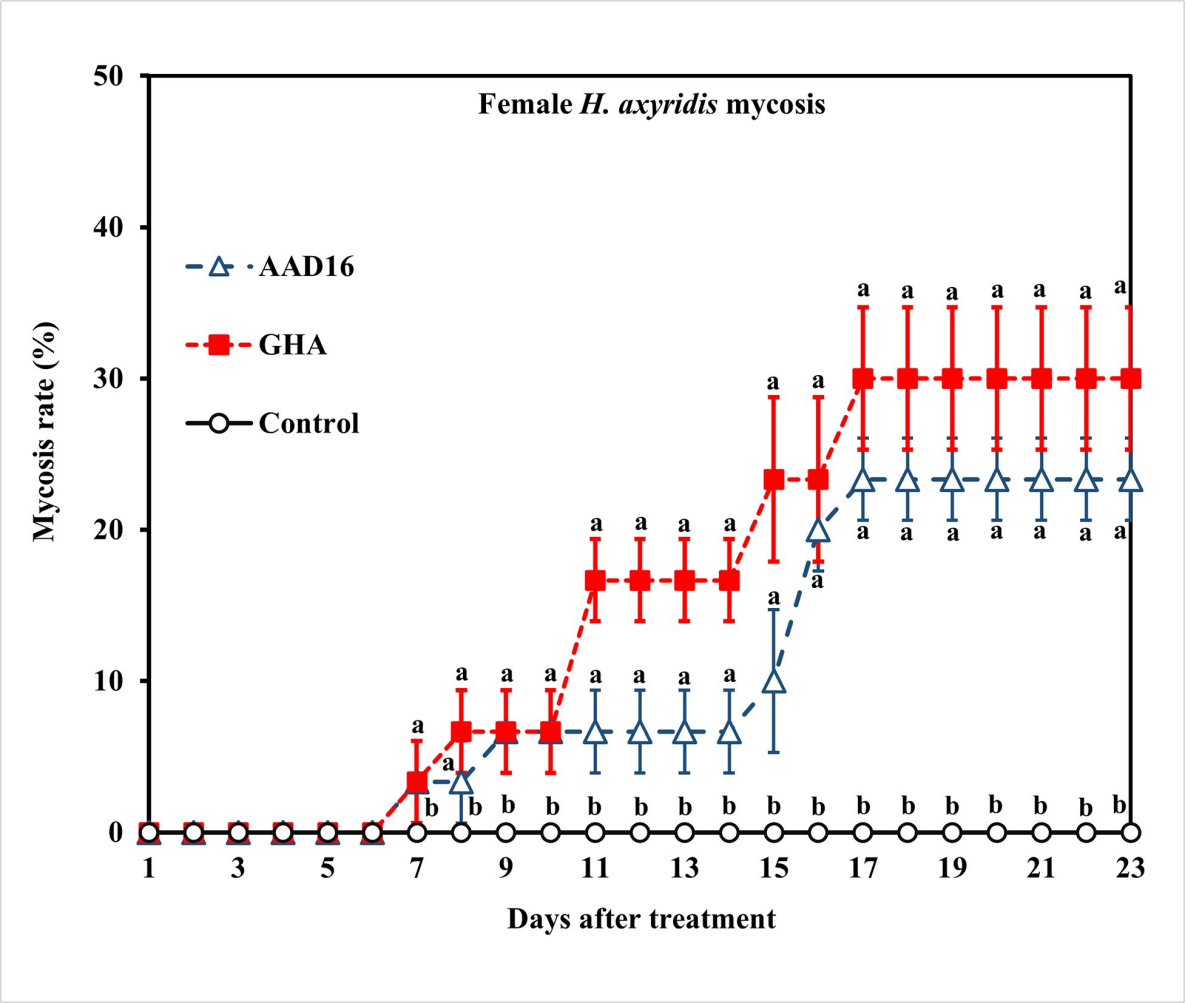
Mycosis rates of adult females of *H*. *axyridis* exposed to 1×10^8^ conidia/ml after exposure for 24 h to different entomopathogenic fungi. Means followed by the same letter are not significantly different (Tukey studentized range HSD test, α = 0.05).

For female adults of *H*. *axyridis*, there were significant differences in ST_50_ values between the AAD16 strain (109.91 h, *χ*^*2*^ *=* 134.95, *df =* 21, *P* < 0.001) and the GHA strain (165.33 h, *χ*^*2*^ *=* 131, *df =* 21, *P* < 0.001) (Table 2). The mortality rate of females was 100% for AAD16 and 97% for GHA, compared to 97% in the control over the entire period of observation (Fig 3). Survival analysis for female adults of *H*. *axyridis* exposed to entomopathogenic fungi showed no significant differences among treatments (*χ*^*2*^ *=* 1.99, *df =* 2, *P =* 0.368) (Fig 3). The mycosis rate for female adults of *H*. *axyridis* showed significant differences between two fungus treatments (Treatment: *F =* 79.35, *df =* 1, 22, *P <* 0.001; Time: *F =* 9.80, *df =* 22, 92, *P <* 0.001; Interaction: *F =* 1.88, *df =* 22, 92, *P <* 0.019) (Fig 4).

**Table 2:** Comparison of fungal efficacy against adult females of *H*. *axyridis* exposed to 1×10^8^ conidia/ml at 1μl per insect via topical application method (n = 30).

Treatment	ST_50_	95% CI	Slope ± SE	*χ*^*2*^ (df)
*B*. *bassiana* AAD16	109.91a	94.31–124.80	5.10 ± 0.43	134.95 (21)
*B*. *bassiana* GHA	165.33b	145.25–184.70	5.24 ± 0.45	131.00 (21)
Control	96.33a	79.79–112.05	4.27 ± 0.40	109.53 (21)

ST_50_ values followed by the same letters are not significantly different among treatments at the 95% CI.

ST_50_, Median survival time; CI, Confidence interval.

**Fig 5:**
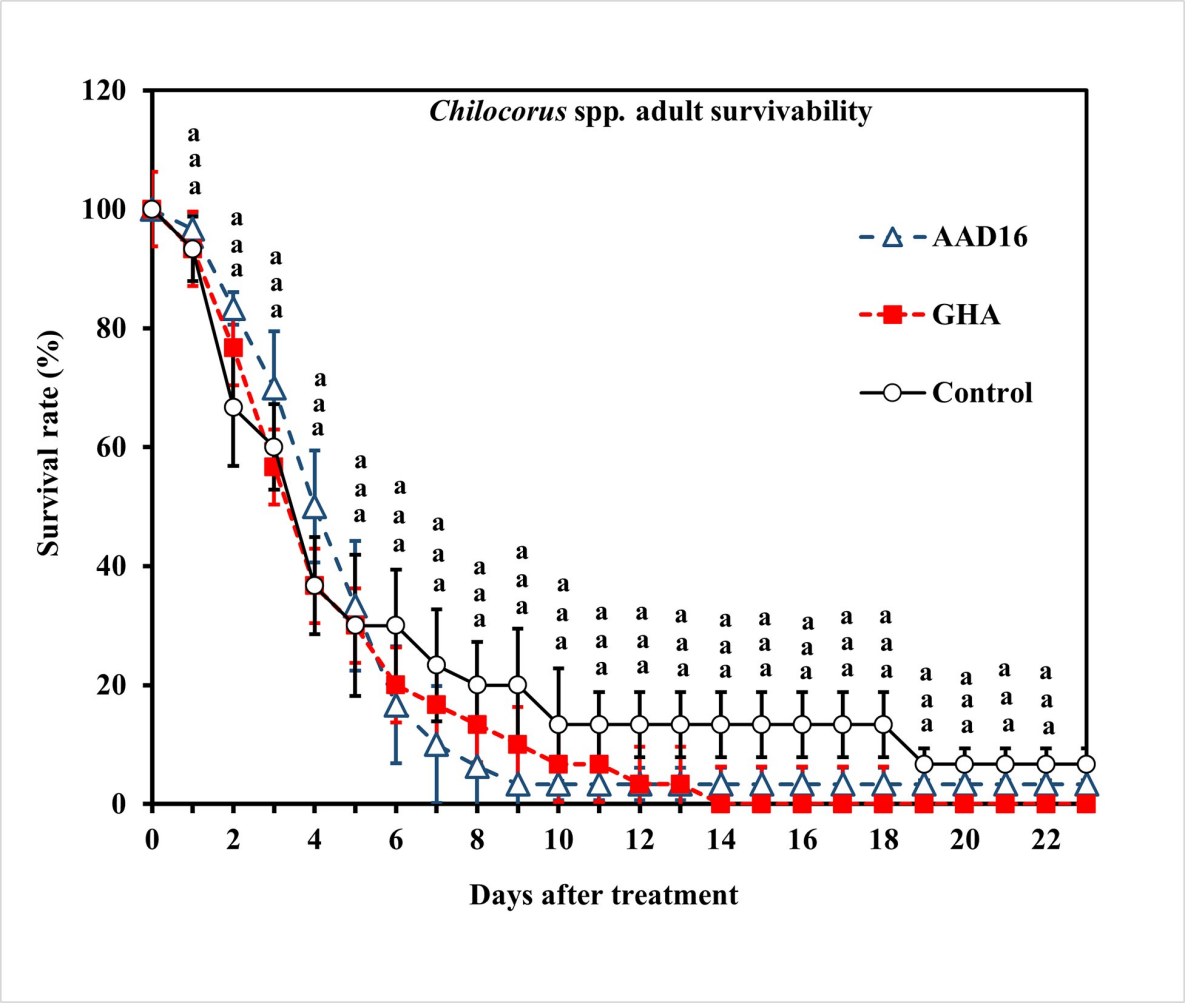
Survival (%) of adults (mixed sexes) of *Chilocorus* spp. exposed to 1×10^8^ conidia/ml after exposure for 24 h to different entomopathogenic fungi. Means followed by the same letter are not significantly different (Tukey studentized range HSD test, α = 0.05).

**Fig 6:**
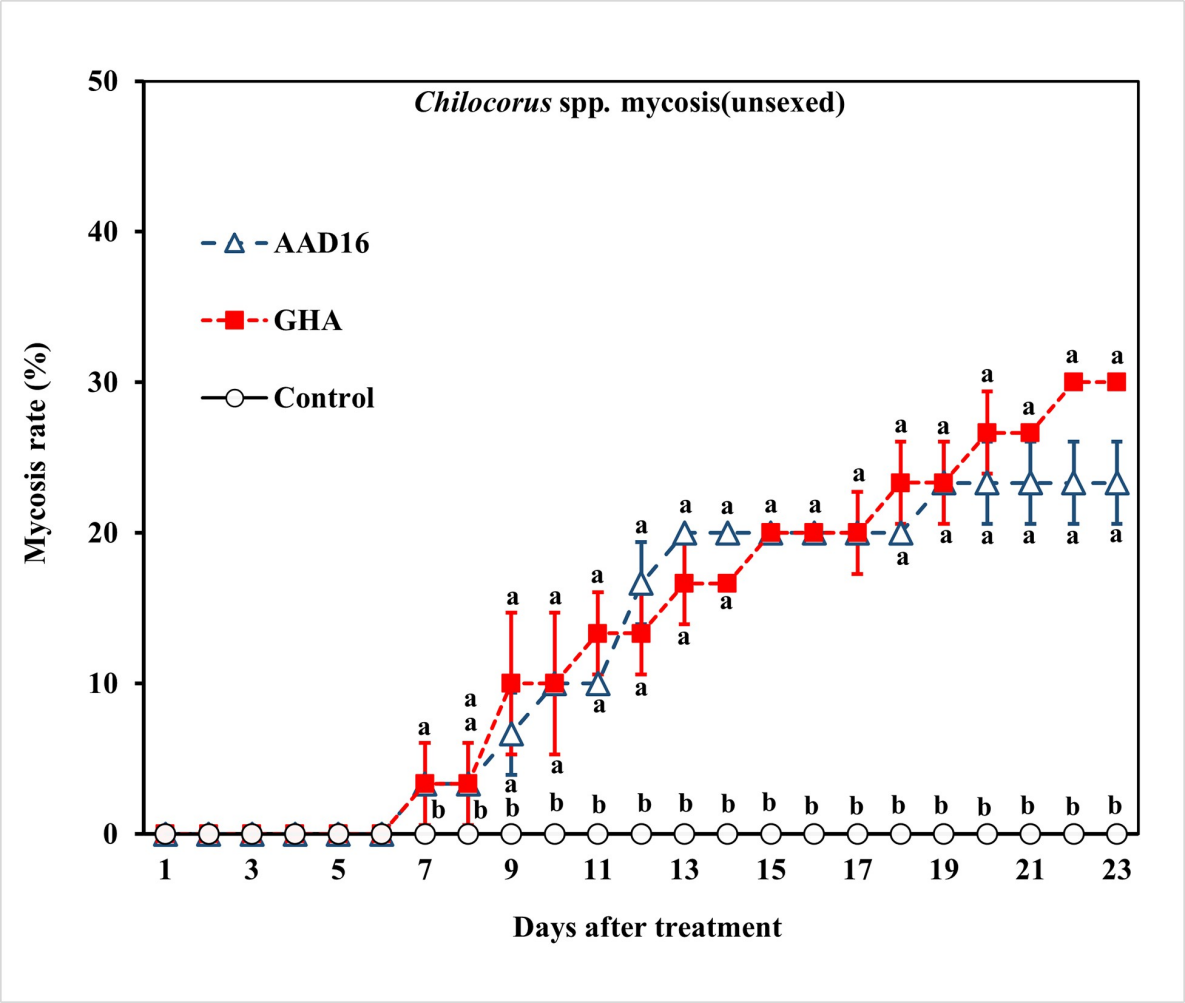
Mycosis rates of adults (mixed sexes) of *Chilocorus* spp. exposed to 1×10^8^ conidia/ml after exposure for 24 h to different entomopathogenic fungi. Means followed by the same letter are not significantly different (Tukey studentized range HSD test, α = 0.05).

For mixed sex adults of *Chilocorus* spp., the ST_50_ value caused by strain AAD16 was 88.96 h (*χ*^2^ = 142.70, *df* = 21, *P* < 0.001), which was not significantly different from that of the GHA strain, which was 79.31 h (*χ*^2^ = 99, *df* = 12, *P* < 0.001) (Table 3). The survival analysis conducted on adults of *Chilocorus* spp. exposed to entomopathogenic fungi showed no significant differences among treatments (*χ*^*2*^ *=* 1.99, *df =* 2, *P =* 0.368) (Fig 5). Mycosis rates were also significant between *B*. *bassiana* AAD16 and GHA (Treatment *F =* 6.00, *df =* 1, 22, *P <* 0.001; Time *F =* 38.77, *df =* 22, 92, *P <* 0.016; Interaction *F =* 0.86, *df =* 22, 92, *P <* 0.644) (Fig 6).

**Table 3:** Comparison of fungal efficacy against unsexed adults of *Chilocorus* spp. exposed to 1×10^8^ conidia/ml at 1μl per insect via topical application method (n = 30).

Treatments	ST_50_	95% CI	Slope ± SE	*χ*^*2*^ (df)
*B*. *bassiana* AAD16	88.96a	76.65–100.73	5.94 ± 0.49	142.70 (21)
*B*. *bassiana* GHA	79.31a	67.85–90.14	5.90 ± 0.59	99.50 (12)
Control	99.60a	82.74–115.66	4.24 ± 0.40	111.62 (21)

ST_50_ values followed by the same letters are not significantly different among treatments at the 95% CI.

ST_50_, Median survival time; CI, Confidence interval.

### Larval bioassay for both lady beetle species

**Fig 7:**
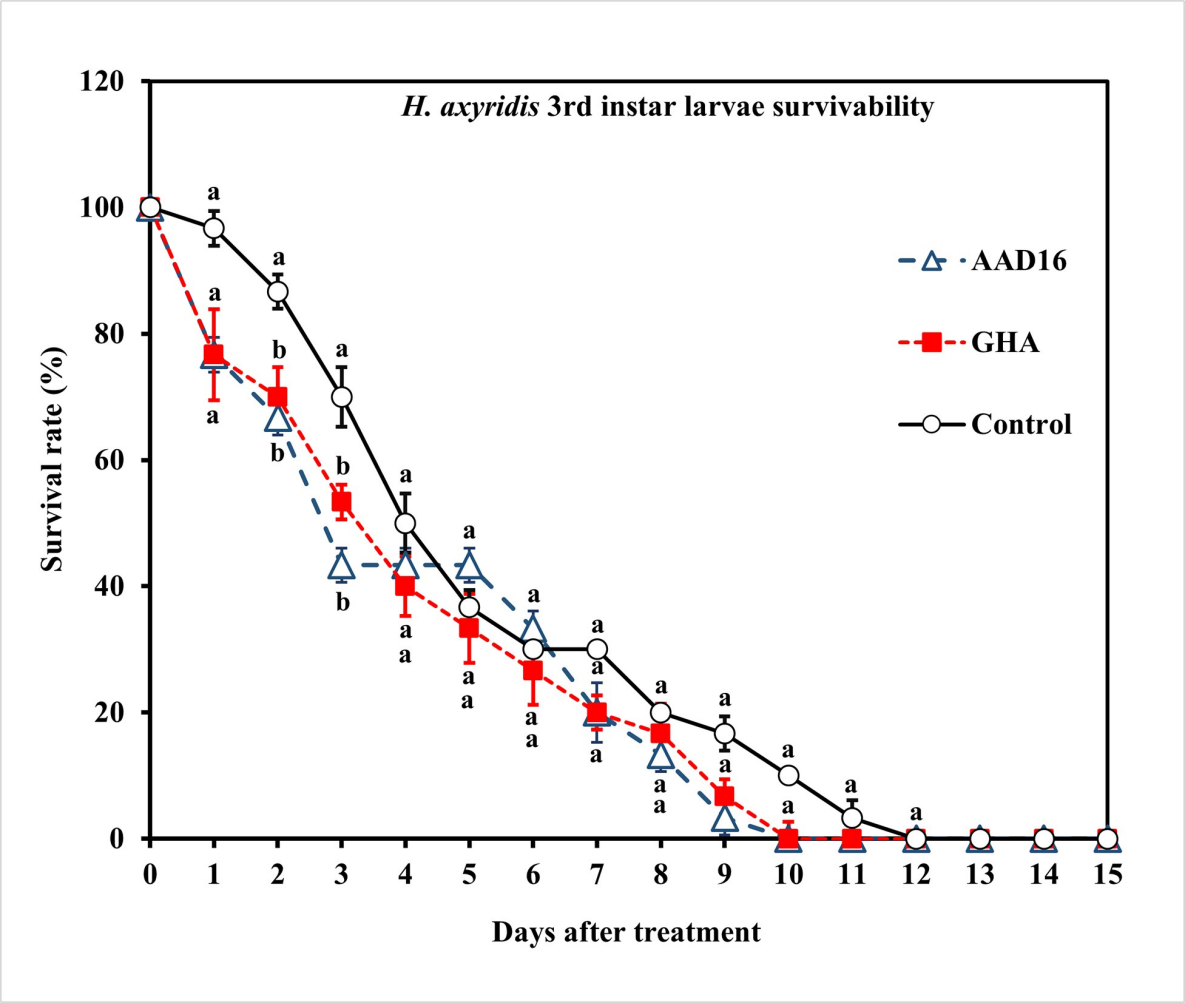
Survival (%) of the 3rd instar larvae of *H*. *axyridis* exposed to 1×10^8^ conidia/ml after exposure for 24 h to different entomopathogenic fungi. Means followed by the same letter are not significantly different (Tukey studentized range HSD test, *α* = 0.05).

**Fig 8:**
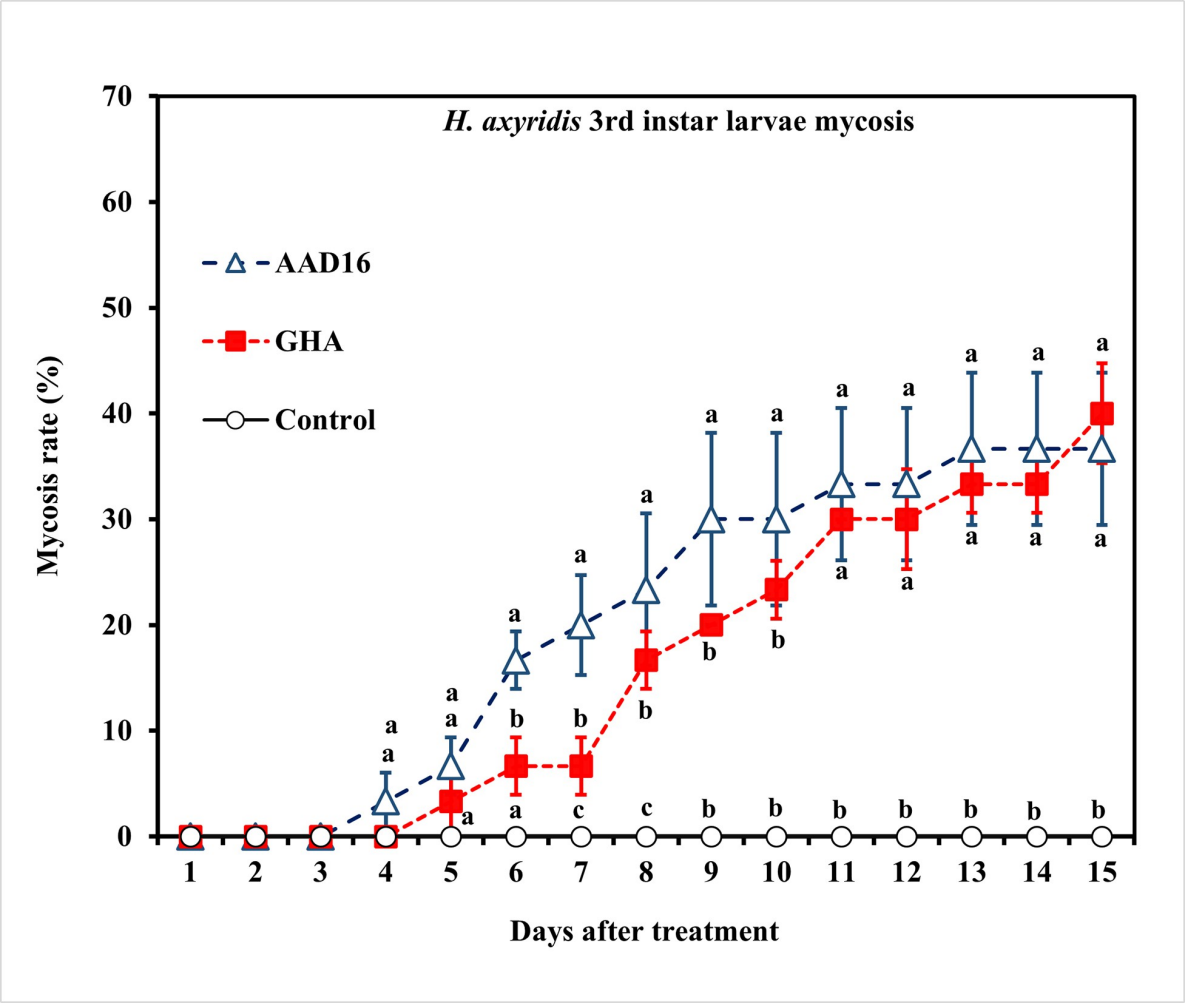
Mycosis rates of the 3rd instar larvae of *H*. *axyridis* exposed to 1×10^8^ conidia/ml after exposure for 24 h to different entomopathogenic fungi. Means followed by the same letter are not significantly different (Tukey studentized range HSD test, *α* = 0.05).

The virulence of the two entomopathogenic fungal strains to 3rd instar larvae of *H*. *axyridis* was similar, with the ST_50_ value of AAD16 being 67.88 h (*χ*^*2*^ = 31.09, *df* = 8, *P* < 0.001) and that of GHA being 72.10 h (*χ*^*2*^ = 55.82, *df* = 9, *P* < 0.001), which were not significantly different (Table 4). Repeated measures ANOVA showed for *H*. *axyridis* larvae, there were significant differences among treatments (Treatment *F =* 16.08, *df =* 2, 14, *P <* 0.001; Time *F =* 191.35, *df =* 14, 90, *P <* 0.001; Interaction *F =* 2.41, *df =* 28, 90, *P* = 0.009) (Fig 7). No significant differences in the mycosis rate were observed between two fungus treatments for larvae of *H*. *axyridis* (Treatment: *F =* 0.56, *df =* 1, 14, *P =* 0.456; Time: *F =* 10.97, *df =* 14, 60, *P <* 0.001; Interaction: *F =* 1.08, *df =* 14, 60, *P =* 0.397) (Fig 8).

**Table 4:** ST_50_ values of different fungal strains for 3rd instar larvae of *H*. *axyridis* exposed to 1×10^8^ conidia/ml at 1μl per insect via topical application method (n = 30).

Treatment	ST_50_	95% CI	Slope ± SE	*χ*^*2*^ (df)
*B*. *bassiana* AAD16	67.88a	46.45–87.32	4.21 ± 0.75	31.09 (8)
*B*. *bassiana* GHA	72.10a	57.87–85.49	4.06 ± 0.54	55.82 (9)
Control	99.76b	87.69–111.36	6.81 ± 0.70	94.62 (10)

ST_50_ values followed by the same letters are not significantly different among treatments at the 95% CI.

ST_50_, Median survival time; CI, Confidence interval.

**Fig 9:**
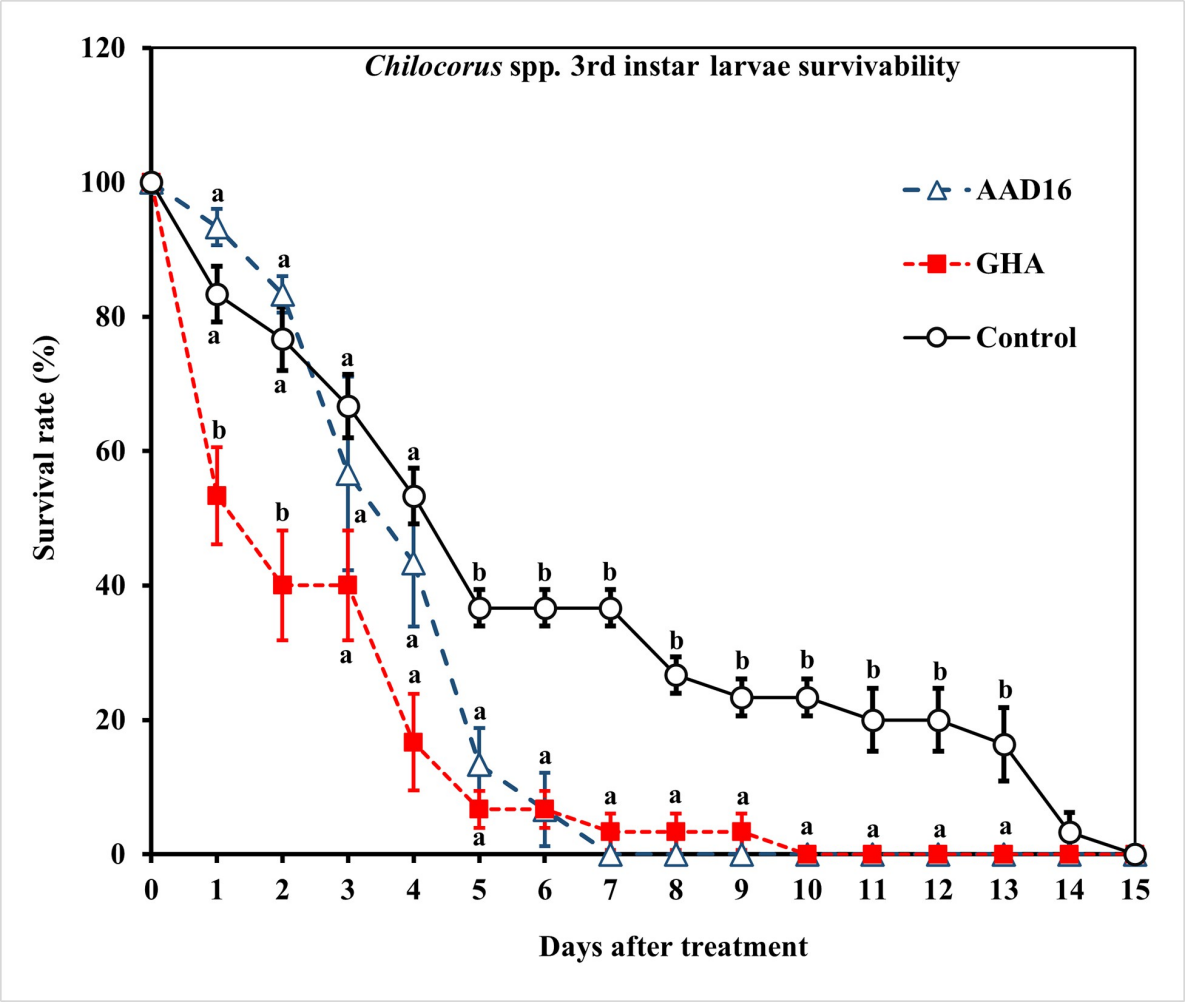
Survival (%) of the 3rd instar larvae of *Chilocorus* spp. exposed to 1×10^8^ conidia/ml after exposure for 24 h to different entomopathogenic fungi. Means followed by the same letter are not significantly different (Tukey studentized range HSD test, *α* = 0.05).

**Fig 10:**
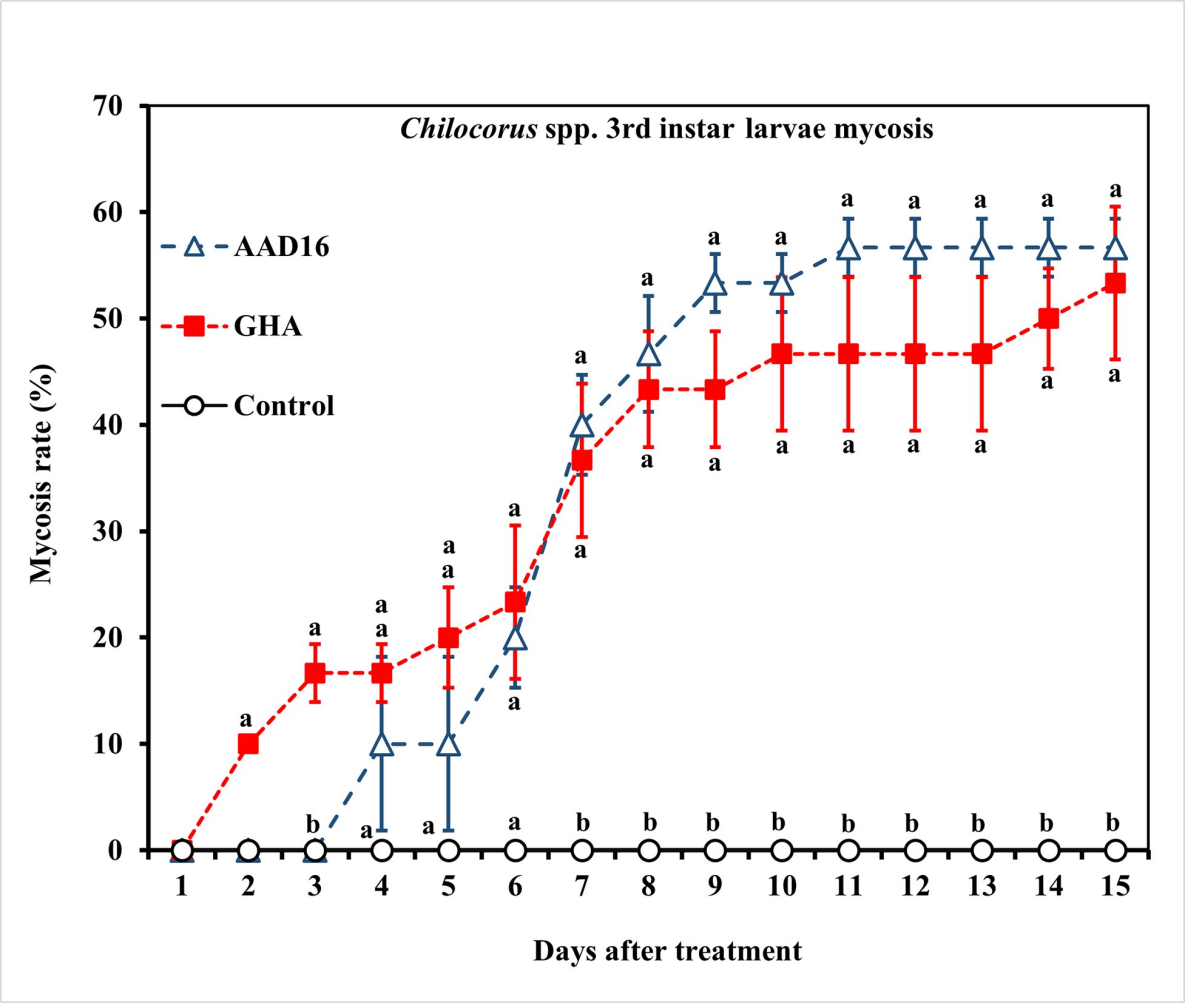
Mycosis rates of the 3rd instar larvae of *Chilocorus* spp. exposed to 1×10^8^ conidia/ml after exposure for 24 h to different entomopathogenic fungi. Means followed by the same letter are not significantly different (Tukey studentized range HSD test, *α* = 0.05).

**Fig 11:**
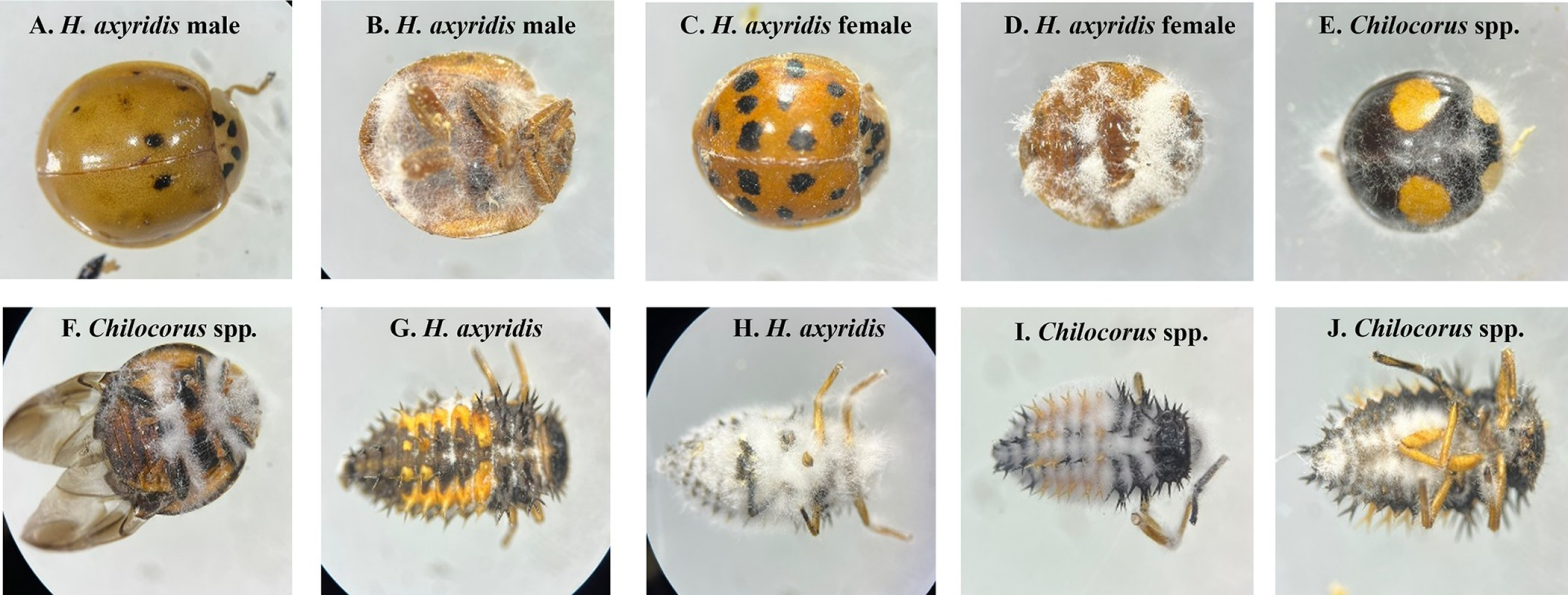
Mycosis symptoms in ladybird beetles caused by *B*. *bassiana* strains AAD16 (A, C, E, G, I) and GHA (B, D, F, H, J).

For *Chilocorus* spp. larvae, the ST_50_ value of AAD16 was 78.88 h *(χ*^*2*^ = 47.53, *df* = 8, *P* < 0.001), while that of GHA was 34.20 h (*χ*^*2*^ = 32.68, *df* = 7, *P* < 0.001), which differ significantly. (Table 5). The ST_50_ for both the AAD16 and GHA strains were significantly different from that of the control, which died off more rapidly than the fungal treated beetles (Table 5) (Treatment: *F =* 22.84, *df =* 2, 14, *P <* 0.001; Time: *F =* 22.27, *df =* 14, 90, *P <* 0.001; Interaction: *F =* 9.08, *df =* 28, 90, *P <* 0.001) (Fig 9). There were no significant differences in mycosis rate between two fungus treatments (not including the control, which does not cause mycosis) (Treatment: *F =* 0.46, *df =* 1, 14, *P =* 0.502; Time: *F =* 23.12, *df =* 14, 60, *P <* 0.001; Interaction: *F =* 0.93, *df =* 14, 60, *P* = 0.533) (Figs 10 and 11). The mycosis rate was 63% in *B*. *bassiana* AAD16 and 60% in *B*. *bassiana* GHA (Fig 10).

**Table 5:** ST_50_ values of different fungal strains for 3rd instar larvae of *Chilocorus* spp. exposed to 1×10^8^ conidia/ml at 1μl per insect via topical application method (n = 30).

Treatments	ST_50_	95% CI	Slope ± SE	*χ*^*2*^ (df)
*B*. *bassiana* AAD16	78.88b	64.13–92.47	8.93 ± 1.29	47.53 (13)
*B*. *bassiana* GHA	34.20a	23.62–43.53	3.57 ± 0.62	32.68 (7)
Control	96.51c	80.33–112.03	4.28 ± 0.48	79.84 (13)

ST_50_ values followed by the same letters are not significantly different among treatments at the 95% CI.

ST_50_, Median survival time; CI, Confidence interval.

## Discussion

The impact of entomopathogenic fungi (EPF) upon arthropod natural enemies, specifically the predaceous Coccinellidae, is not well understood [35]. The process of choosing biological control agents for pest management programs typically involves considering factors such as host range, geographic distribution, fecundity, and voltinism. However, the evaluation of their susceptibility to insect pathogens native to the region is rarely integrated into this decision-making process. Specifically, for the effective establishment of natural enemies in new environmental settings—whether they involve different geographical locations or crop cultivation—it is crucial to assess the potential risk posed by endemic pathogens [46]. EPFs are infrequently found infecting coccinellids (ladybird beetles) under natural conditions [47], and studies have shown that natural fungal infections in these beetles rarely exceed 20% [47, 48]. Among EPFs, *B*. *bassiana* stands out as a generalist pathogen capable of infecting both plant-feeding and predatory coccinellids [49, 50].

Interspecific interaction among natural enemies is one of the important factors determining effectiveness of biological control programs [51]. However, the interactions between *B*. *bassiana* and coccinellids are rarely known, although the interactions between EPFs and some other types of natural enemies (parasitoids and predators) have been studied [52–55]. Applying the EPFs that are compatible with other natural enemies could result in higher control efficacy without increasing risk to non-target organisms and with reduced use of conventional insecticides [56].

In the present study, we investigated the susceptibility of *H*. *axyridis* and *Chilocorus* spp. lady beetles exposed to two EPFs (*B*. *bassiana* AAD16 and GHA strain) under laboratory conditions. We found significant differences in ST_50_ values between the AAD16 and GHA strains when tested against adult females of *H*. *axyridis*. However, there was no significant difference observed in adult males of *H*. *axyridis* and *Chilocorus* spp. during the 23 days of our trial (Tables 1–3).

Although the level of difference in mortality between the two ladybird species within a fungal strain was not significant, it is certainly worth further exploration. Several studies have illustrated the physiological susceptibility of coccinellids to *B*. *bassiana* [19]. Cottrell and Shapiro-Ilan [19] specifically emphasized the differing susceptibility of a native and an exotic coccinellids to an isolate of *B*. *bassiana* originating from the native coccinellid. The exotic coccinellid, *H*. *axyridis*, demonstrated lower susceptibility to *B*. *bassiana* compared to the indigenous coccinellid, *Olla v-nigrum* Mulsant (Coleoptera: Coccinellidae). Cagan and Uhlik [57] demonstrated that *B*. *bassiana* strains isolated from *Ostrinia nubilalis* were pathogenic to *Coccinella septempunctata* and *Propylea quatuordecimpunctata*. However, they suggested that field susceptibility of these ladybirds would likely be lower due to reduced contact with the pathogen in natural settings. Our findings showed that neither the coccinellids we tested were affected in either mortality or mycosis by the EPF strains assessed here.

*H*. *axyridis* has shown great resistance to entomopathogenic nematodes and the entomopathogenic fungi *B*. *bassiana* [26]. The resistance of *H*. *axyridis* to *B*. *bassiana* involves potential mechanisms such as melanin production at infection sites [58] or defensive chemicals [59–61]. Other insects facing pathogen-rich environments, such as rat-tailed maggots of the drone fly thriving in polluted aquatic habitats like liquid manure storage pits [62], and the burying beetle *Nicrophorus vespilloides* Herbst, which feeds and reproduces on cadavers [63], also exhibit heightened pathogen resistance attributed to a diverse array of antimicrobial peptides (AMPs). Nevertheless, both fungi we tested were more pathogenic to the larval stage than the adult in both species of lady beetles.

For successful integration of EPFs into integrated pest management (IPM) programs, mycoinsecticides must be virulent to the target pests [42, 64, 65] but have only minimal negative effects on non-target organisms [66]. Indeed, the non-target effect of different EPFs on natural enemies has been assessed by many previous studies. Ullah et al. [67] tested the virulence of *Isaria fumosorosea* Wize and *B*. *bassiana* against a reduviid predator and reported no significant impact upon predation and survival rate.

Huang et al. [68] reported that various concentrations of *B*. *bassiana* had no significant effect on biological parameters of the coccinellid *Prynocaria cogener* (Billberg). Similarly, in other laboratory investigations, *B*. *bassiana* was found not to be pathogenic to some beneficial arthropods, including *Apis mellifera* L., *Chrysoperla rufilabris* Burmeister, *Orius insidiosus* Say, *Hippodamia convergens* Guérin-Méneville, *H*. *axyridis*, and *Coleomegilla maculata* De Geer [69]. Harwood et al. [70] reported very low infection rates of *Hesperomyces virescens* Thaxter on coccinellids.

Until now, no entomopathogenic fungus has been studied to determine its effect on *Chilocorus* spp. *B*. *bassiana* AAD16 was isolated from *A*. *dichotoma*, it is comparatively less harmful against both species of coccinellids tested compared to the commercial *B*. *bassiana* GHA. Therefore, the two predatory coccinellids would be compatible with the AAD16 strain. In the future, the mechanism underlying the compatibility of these two coccinellid beetles with *B*. *bassiana* AAD16 and GHA should be determined, and in addition, those compatibilities should be verified under field conditions.

## Supporting information

S1 Data Raw data_male *H*. *axyridis*, raw data_female *H*. *axyridis*, raw data_adult *Chilocorus* spp., raw data_*H*. *axyridis* larvae, raw data_*Chilocorus* spp. larvae. (XLSX)
