# Correction: Laboratory risk assessment of *Beauveria bassiana* AAD16 on two species of ladybird beetle

**DOI:** 10.1371/journal.pone.0320099

**Published:** 2025-03-17

**Authors:** Md. Rajib Hasan, Md. Rasel Raju, Un Taek Lim

The Funding statement for this paper is incorrect. The correct Funding statement is as follows: This work is supported by Korea Institute of Planning and Evaluation for Technology in Food, Agriculture, and Forestry (IPET) through Agricultural Machinery/Equipment Localization Technology Development Program, funded by Ministry of Agriculture, Food, and Rural Affairs (MAFRA)(321054-05-2-HD020).
